# From Bill Shankly to the Huffington Post: How to Increase Critical Thinking in Experimental Psychology Course?

**DOI:** 10.3389/fpsyg.2016.00538

**Published:** 2016-04-19

**Authors:** Emilie Lacot, Geoffrey Blondelle, Mathieu Hainselin

**Affiliations:** ^1^CRP-CPO, EA 7273, Université de Picardie Jules VerneAmiens, France; ^2^Centre d'Activité de Génétique Clinique et d'Oncogénétique, Centre Hospitalier Universitaire Amiens PicardieAmiens, France

**Keywords:** critical thinking, pedagogy, belief, reviewing process, authority

## Abstract

Although critical thinking and source checking are basic prerequisites to become a psychologist, or a scientist, it is usually difficult to have students interested in experimental methods courses. Most first year students are tempted not to attend these courses. Such behaviors are reinforced by arguments that “everybody is different” and “people are not numbers.” Consequently, students have difficulties to develop source and evidence checking skills, and may be more prone to believe in any supposed expert. This paper presents two ways to involve students during lectures and seminars. The first method consists in presenting, during the initial lecture of the year, a fake scientific concept which students will believe as true. This phenomenon is called the “Bill Shankly syndrome” and it only exists if someone believes that the information is given by a serious lecturer, presenting oneself as a world-class researcher. The second method consists in training students to become reviewers using evidence checking of a mainstream media article which promises scientifically proven ways to be happy. The use of these methods may stimulate students' interest in research methods and its practical applications from week one.

## Introduction

Psychology students, especially first year university students in France, attend their first lessons with many beliefs about psychology. Most of them think that it is not a scientific field, and are thus surprised with the number of courses on neuroscience, statistics and methodology. At the very beginning, students are confronted with the different steps of the scientific method and discover the mean of a sample, which is frequently and wrongly associated with the mean of a population. These concepts are usually in contradiction with their own conceptions such as “everybody is different” and “people are not numbers” (Malekoff, 2008). Due to these misconceptions, students may encounter difficulties in understanding or seeing the usefulness of such courses (Castro Sotos et al., 2007; Gigerenzer et al., 2007).

Moreover, for many reasons, it is hard for them to develop critical thinking or any experimental methodology skills. In high school, and more specifically in France, psychology is a tiny part of the Philosophy course, only studied through Freud's theories of psychoanalysis (Lieury, 2014). Although, APA have a resource manual for psychology teaching including critical thinking (APA, 2015), students have barely been trained for critical thinking, as most part of their schooling is based on a passive listening model (e.g., Paul, 1992). Wegwert (2014, 141) highlighted that “fear is a powerful presence in schools, in teacher education, and in teacher identity” and consequently in students daily life. Because of the perceived power of lecturers, which can intimidate students, they feel compelled to integrate knowledge without checking its truthfulness. Consequently, students may be more prone to believe in any authority figure and are less inclined to show a critical mind (Wegwert, 2014). In the same vein, Reeve (2009), Skinner and Belmont (1993), highlight that the same phenomenon occurred if lecturers do not adopt an autonomy-supportive style. In our sense, these facts inhibit the development of great psychologists or scientists. However, a growing literature underlines that psychologists, scientists and thus lecturers can, due to a lack of evidence-based practice, make inappropriate inferences. For example, in their recent paper, Lilienfeld et al. (2014) highlight four barriers to scientific thinking: naïve realism, confirmation bias, illusory causation, and illusion of control. Thus, using concrete examples of inappropriate inferences in patients' treatment could help students identifying those biases. For example confirmation bias: I think I am a good therapist so patients have improved with me, when in fact it may come from an external event. The purpose is to show the many steps needed for any scientific reasoning, as well as for psychotherapies evaluation, and for possible clinical thinking.

We propose two different methods to engage first year psychology students and promote critical thinking. The first method, which must be used during the initial experimental methods lecture, consists of presenting a false scientific concept: the “Bill Shankly syndrome,” presented in the first part of this article. This method is supposed to sharpen the critical mind of the student and to lead them to make their own scientific research. However, first year students of psychology tend to search for information on the Internet without verifying the scientific quality of the sources. To change this behavior, we have used, during seminars, a participative method based on the criticism of research supposedly defined as scientific. This second method, described in the second part of this article, allows the students to distinguish between a scientific source and a non-scientific source and also to criticize the methodology used.

## The Bill Shankly syndrome: A serious joke for a lesson

We propose a method that was used with first year students of psychology at Jules Verne University of Picardy. This method consists in presenting a scientific concept: the “Bill Shankly syndrome” during the initial lecture of the year. However, the success of this method benefits from a specific context (i.e., the lecturer must have some authority). Before the presentation of the concept, the university lecturer introduces oneself to the students. The lecturer boasts very seriously about his career, asking students to call him “Doctor” (which is very unusual in France if you are not a Medical Doctor) due to his PhD, explain that he is an Associate Professor at the Department of psychology, a trained neuropsychologist, works with high-level research teams in different countries, publishes articles and is asked for his expertise (i.e., as a reviewer) for international scientific journals. Although, this is a standard resume for an Associate Professor, students are unaware of it. Then, this peremptory introductory speech takes place in an unusually silent amphitheater. This assertive presentation is determinant in the Bill Shankly syndrome. Immediately, the PowerPoint lecture starts, with the classical pavlovian-writing behavior (students blindly copying after the presentation of each slide) and moving on to the next slide. Note that this type of presentation is important too. In fact, the use of PowerPoint animations enable a chronological presentation (i.e., scrolling the sentences one by one), better note taking and, as all lecturers hope, a better understanding of courses (Schmaltz and Enström, 2014). The concept that we arbitrarily called the Bill Shankly syndrome, because the last author (MH) is a Liverpool F.C. fan, is presented as a main concept in psychology. The lecturer expresses it as naturally and seriously as possible. It is written on slides and read out to the students that the syndrome consists in believing that any truth is the truth because this truth is named, expressed, illustrated as a scientific truth. This definition is followed by a reference to a fake scientific reference “Shankly et al., 1959” (see Supplementary Material for the slide), with a fake concept. The only real thing in this part is the Bill Shankly black and white picture. The definition remains deliberately vague to reinforce social influence (see below). Above all, choosing a 1960's Scottish football manager allow us to emphasize that anything, including old sports references, can be seen as scientific if students do not improve their critical thinking.

The concept that was introduced was clearly flawed, so that someone with critical thinking skills should question the validity of such a claim. To believe that everything is true because someone says so should be an aberration for psychologists, scientists or even for students. In principle, critical thinking involves questioning concepts and existing theories. However, our example highlights that the majority of the first year students agree with this concept. In the past couple of years, about 1000 students attended this course. They took notes without one single objection, and none of them asked any questions. This silence can be interpreted as the students' idea of university lecturers as having a great deal of knowledge. Note that these effects (i.e., silence and note taking without questioning) can be increased by informational or normative social influences and by conformity behaviors (students might assume the actions of others in an attempt to reflect correct behavior for a given situation). Indeed, if the majority of students write in silence without questioning courses, the others are more likely to do the same. It highlights the strong influence of peer group, the compliance and the conformity, particularly in a new situation with possible anxiety (Guimond, 1997; Cialdini and Goldstein, 2004). Altogether, this is a great place to discuss informational social influence with students.

Following the presentation of the Bill Shankly syndrome, the university lecturer explained that this concept is false. His speech was supported by a new sentence appearing on the slide: “the Bill Shankly syndrome” obviously does not exist…unless you believe it…and thus you become a victim. Then the lecturer explained that William (Bill) Shankly (1913–1981) was a Scottish footballer and manager of Liverpool (Peace, 2014). During the time of these explanations, it was interesting to note that the majority of students were still writing down the PowerPoint's sentences. Nevertheless, some of them understood the joke lesson and then they initiated discussion about the lecturer's speech. Indeed, despite his position as a lecturer/expert, all content should be supported by expert scientific support. At this point, the fake concept was disclosed and the lecture started again from the beginning with an as-normal-as-possible presentation of the lecturer and the course. The pedagogical aim, besides the academic message, is to generate in students the feeling that they can be victims of several cognitive biases and more largely they can be victims of social phenomena. Indeed, due to the social status of the university lecturer, students believe his speech without questioning the situation or the contents. Thus the message given here is: “enhance your critical thinking: don't believe everything you listen or read whoever the speaker/author is.” Our aim is that students keep an open and critical mind. This requires searching for scientific information outside the courses. However, in the same way that students believe in the lecturer, they can also think that all Internet retrieved information is true and scientific, particularly if there is an expert cited (i.e., some students have cited blog posts as a scientific reference because the blogger claimed, incorrectly, to be an expert). The lecturer must warn them against false sources, and to check for original scientific source rather than just blindly believing indirect sources (i.e., self-proclaimed expert) instead of fact checking. During this first lecture, the experimental method, scientific journals standards, including peer-review process, are presented. As a follow up to this exercise, students are presented with an opportunity to seek out valid scientific sources.

## First reviewing experience: First disappointment

Like the first method presented, the second one is also used with the first year students of psychology in Jules Verne University of Picardy during seminars, always occurring after the first lecture with the Shankly effect. Thus, students are already aware of the importance of reconsidering the lecturer's discourse content. They also know the need to verify supposedly scientific knowledge. Another important point is that this method must be used during seminars. As there are fewer students in seminars (taking place in classroom, 30–40) than in lectures (taking place in a lecture hall, 250–300), students are more likely to speak in public, which is usually a difficult exercise for them.

This second method consists in training students to become reviewers using evidence checking. More specifically, three points should be broached: (i) distinguish a scientific source (i.e., peer-reviewed journal) vs. a non-scientific source (i.e., without peer-review), (ii) criticize the proposed hypotheses, and (iii) propose another experiment to check, replicate or go further. In this way and after a brief presentation, the lecturer starts the PowerPoint course based on an article from the Le HuffPost (2013). It proposes 13 tricks scientifically proved to make people feel happy; each trick is displayed in Table 1. Tricks are seen one by one with the students.

**Table 1:** **Examples of tricks and justification to be happy from the studied article, with found references and possible criticizes**.

**Tricks**	**Justifications**	**References**	**Criticizes**
1. Make a gift.	According *a study*, kind souls are happy.	Dunn et al., 2008	Scientific article in *Science*. Possible limits pinpointed because of financial resources of the participants.
2. Count his/her moments of happiness.	*A professor* at the University of Pennsylvania has demonstrated […] were happier than the average.	Seligman, 2012	Book. Non-scientific. What does mean average? Compared to whom?
3. Live new experiences.	Nevertheless, *researches* that get out of the daily routine made happy.	MSN Lifestyle.	Non-scientific.
4. Anticipate good times.	None	Blog.	Non-scientific.
5. See the life in blue.	Sussex University scientists have shown that […].	Interview of the concerned scientists retrieved from the website DailyMail, Macrae, 2009.	Non-scientific. Possibility of conflicts of interests.
6. Define targets.	[…] according to the psychologist Richard Davidson.	Jackson et al., 2000	Scientific article but it does not match with the tricks (different topic).
7. Stop always wanting to be right.	The writer and author Deepak Chopra recommends to his readers to remain neutral […].	Interview of Deepak Chopra, FoxNews, 2011	Non-scientific. Does not match with the tricks.
8. Go to church.	[…] according to the Melbourne University.	DailyMail, 2010b	Non-scientific. The study is not published.
9. Sleep at least 6 hours a night.	In a study […] assessed their level of happiness on a scale varying from 1 to 5 […].	DailyMail, 2010a	Non-scientific. What does mean a level of happiness? Have we the same definition?
10.20min in means of transport.	British scientists advise to limit the path that leads to work […].	DailyMail, 2010a	Non-scientific. Some factors will be beyond the person's control.
11. Have at least 10 friends.	Authentic friend can be counted on fingers. This expression is familiar but for scientists […].	Website DailyMail, Macrae, 2008	Non-scientific. How to define a friend as being a real friend?
12. Keep it positive even at wrong times.	According to a psychologist […].	Website Howstuffworks, Layton, 2009	Non-scientific. Use of words as “it seems.”
13. Don't forget be in love!	None.	None.	The entire trick!

First, only the trick and the original picture in the article were presented. Students were asked to say what they think about the trick and decide how they would validate the claim using source checking and critical thinking. For every trick, the first step is to find the source (scientific journal or not) by clicking on a link. The Huffington Post was chosen because it is a digital media allowing links, and because, except for this particular article, many good scientific popularization articles are available (Eustache, 2014). The aim is to make students check sources as often as possible, not to destroy any journal reputation.

When any limit or lack of scientific reference is underlined, they have to suggest a new experiment to assess the trick validity. This was a first step into scientific methods.

## Perspective

The Shankly effect and the media source checking are engaging exercises to teach experimental methods. After showing some surprise in the first place, students seem to like this new approach to enhance critical thinking (i.e., the Bill Shankly syndrome and its explanation), and use it beyond the specific course.

Although generalization is expected, there are cautions for instructors who are going to use this approach. The first concern is an over-generalization of the “don't believe everything you listen or read, whoever the speaker/author is” to every single point of every lesson. If too many students ask lots of justification questions during the lecture, it might prevent the lecturer from saying everything s/he would have liked to say. In our experience, it might also be passed on to other lessons and lecturers, for whom over-questioning and fact checking could be unusual. To avoid this, we ask for constructive criticism and always give our sources so students can check themselves. While these approaches have worked well for the authors, the evidence is anecdotal and as of yet, limited to students in France. Our methods are however consistent with recent recommendation to develop critical thinking (Schwanz and McIlreavy, 2015). We strongly encourage instructors to try these methods, as well as other engaging methods, to help promote critical thinking among students. Future research is needed to assess the actual efficiency, short and long term, of the Shankly effect.

## Author contributions

All authors listed, have made substantial, direct, and intellectual contribution to the work, and approved it for publication.

### Conflict of interest statement

The authors declare that the research was conducted in the absence of any commercial or financial relationships that could be construed as a potential conflict of interest.
