# Supplementary figures and images for: From Bill Shankly to the Huffington Post: How to Increase Critical Thinking in Experimental Psychology Course?
Source: Front Psychol. 2016 Apr 19;7:538. doi: 10.3389/fpsyg.2016.00538 (PMC4835719; doi:10.3389/fpsyg.2016.00538)

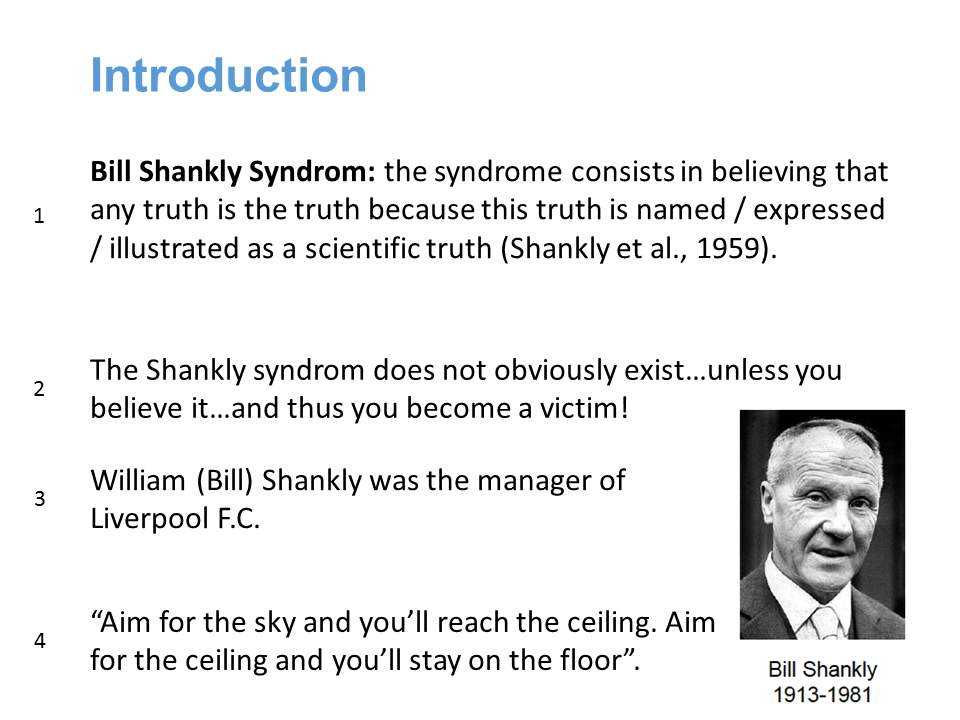

Supplement: Supplementary file 1 [file Image1.PNG]
